# Expression Profiles of Circular RNA in Human Atrial Fibrillation With Valvular Heart Diseases

**DOI:** 10.3389/fcvm.2020.597932

**Published:** 2020-11-20

**Authors:** Xiyu Zhu, Xinlong Tang, Hoshun Chong, Hailong Cao, Fudong Fan, Jun Pan, Dongjin Wang, Qing Zhou

**Affiliations:** Department of Cardio-Thoracic Surgery, Nanjing Drum Tower Hospital, The Affiliated Hospital of Nanjing University Medical School, Nanjing, China

**Keywords:** persistent atrial fibrillation, valvular heart disease, circular RNA, mRNA, regulatory network

## Abstract

Circular RNAs (circRNA) are involved in a variety of human heart diseases, however, circRNA expression profiles and circRNA-miRNA-mRNA regulatory network in human atrial fibrillation (AF) especially with valvular heart diseases (VHD) remain poorly understood. A high-throughput RNA sequencing was used to investigate the differentially expressed circRNAs in left atrial appendage from VHD patients with or without persistent AF. The Gene Ontology (GO) and Kyoto Encyclopedia of Genes and Genomes (KEGG) pathway enrichment analysis were performed to predict the potential functions of the host genes of differentially expressed circRNA and their downstream targets. CircRNA–miRNA-mRNA regulatory network was constructed to identify mechanisms underlying circRNAs. qRT-PCR and sanger sequencing were further performed to validate the results. Compared with sinus rhythm (SR) patients, there were 3094 upregulated and 4472 downregulated circRNAs in AF patients respectively. The expression of 10 most differentially expressed circRNAs (circ 255-ITGA7, circ 418-KCNN2, circ 13913-MIB1, circ 44670-BARD1, circ 44782-LAMA2, circ 81906-RYR2, circ 35880-ANO5, circ 22249-TNNI3K, circ 3136-TNNI3K, circ 56186-TNNI3K) between SR and persistent AF patients were verified by qRT-PCR. In addition, specific back-splicing sites of these circRNAs was confirmed by sanger sequencing. GO and KEGG pathway analysis indicated that cAMP signal pathway and Wnt signal pathway might play important role in the development of AF in VHD patients, which might be affected by circRNAs. This study provided a preliminary landscape of circRNAs expression profiles which are involved in persistent AF due to VHD, and established the possibility for future related researches in this field.

## Introduction

Atrial fibrillation (AF), the most common sustained arrthymia in clinical practice, is becoming a major burden to social health care system due to the high risks of stroke and sudden death (1). The current epidemiological study indicates that the overall incidence of AF has increased to about 1% to 2% of the general population (2). AF itself or various clinical conditions, such as aging, hypertension, valvular heart disease (VHD), smoking and obesity (3) are risk factors in developing AF. Valvular AF is considered as an obsolete concept which is always accompanied with mitral valve stenosis or aortic valve stenosis. However, about 2–31% VHD patients suffered from AF according to the data of RE-LY trial (4). Compared to those AF patients without VHD, stroke and system embolism risk is increased in valvular AF patients (5). Also, high recurrence rate of AF results in a poor prognosis and repeated hospitalization. Left atrial dilatation induced structure remodeling is the basis of AF occurrence in VHD patients, however, the molecular mechanism of the formation and development and recurrence remain poorly understood.

Circular RNA (CircRNA) is a new type of non-coding RNA with regulatory function in specific biologic processes (6). Compared with other types of RNA, circRNA does not have a 5′ end cap and a 3′ end poly (A) tail. It is covalently linked end to end to form a closed loop structure, which makes it less susceptible to degradation by nucleic acid exonuclease RNase R and therefore more stable than other types of RNA (7). CircRNA is highly conservative, and increasing studies show that it plays an important role in the occurrence and development of various diseases (8, 9). High-throughput sequencing results show high enrichment of circRNA in heart tissue (10), further researches have showed circRNAs play important role in different biological process of cardiac hypertrophy (11), myocardial infarction (12, 13), cardiac senescence (14) and atherosclerosis (15). However, the expression profiles of circRNAs and its related downstream target regulation network in valvular AF is still unknown.

In this study, we used ten pairs of matched human left atrial tissues (another 18 pairs of matched tissues were used for verification), analyzed differentially expressed circRNA, miRNA and mRNA using high-throughput whole transcriptome sequencing technology, and constructed a circRNA-miRNA-mRNA network, with a view to providing some references for related researches in this field. The top 10 differentially expressed circRNAs was validated with quantitative real-time reverse transcription polymerase chain reaction (qRT-PCR) and Sanger sequence. These significantly different expressed circRNAs could be related to valvular AF occurrence and maintainess and were explored in a series of atrial tissues.

## Materials and Methods

### Patients and Specimens

This study was conducted in accordance with the Declaration of Helsinki and was approved by the Medical Ethics Committee of Nanjing Drum Tower Hospital, the affiliated hospital of Nanjing university medical school (approval number: 2016-151-01). Written informed consents were obtained from all participants.

Left atrial appendage (LAA) specimens were obtained from 56 VHD patients in our center, among which half of the patients were accompanied with persistent AF (PeAF). Pre-operative 24 h ECG Holter examination was used to make the diagnosis of PeAF. VHD was diagnosed by echocardiography. Propensity score match was used to pair match the patients between PeAF and sinus rhythm (SR) group. The clinical characters of these patients were listed in Tables 1, 2. During the surgery, all the specimens were divided into two parts, of which, one was immediately frozen in liquid nitrogen for further experiments, the other was prepared for Masson's staining. For RNA-seq analysis, 10 pairs of PeAF and SR specimens were used. The other 18 paired specimens were used for qRT-PCR validation.

**Table 1:** Clinical characters of patients included in the RNA-Seq.

	**SR (*n* = 10)**	**AF (*n* = 10)**	***P*-value**
Age (y)	55 ± 8	53 ± 8	0.601
Gender (Male/Female)	5/5	6/4	1.000
BMI (kg/m^2^)	24.5 ± 3.7	23.9 ± 3.3	0.716
**Cardiovascular risk factors**			
Hypertension (*n*)	6	1	0.057
Diabetes mellitus (*n*)	0	0	1.000
CKD (*n*)	0	0	1.000
Smoke abuse (*n*)	1	0	1.000
Alcohol abuse (*n*)	0	0	1.000
Coronary artery disease (*n*)	5	0	0.033
Stroke (*n*)	0	1	1.000
**Transthoracic echocardiography**			
LAD (cm)	4.7 ± 0.8	5.8 ± 0.6	0.002
EF (%)	53.0 ± 9.5	51.5 ± 5.7	0.673
**Mitral valve Carpentier's phenotype (n)**			
II	0	6	
IIIa	8	1	
IIIb	2	2	
Mitral regurgitation with stenosis (*n*)	0	4	
**NYHA class (*****n*****)**			
II	4	0	
III	4	9	
IV	2	1	
BNP (pg/mL)	341.8 ± 528.0	266.8 ± 176.9	0.215
CRP (mg/L)	18.6 ± 40.0	16.1 ± 22.8	0.289
eGFR (%)	97.6 ± 22.1	88.7 ± 25.6	0.428
**Drug therapy (*****n*****)**			
Beta blockers	5	5	1.000
Anticoagulants	2	6	0.085
Digoxin	1	4	0.303
PGI2	4	2	0.628

*BMI, body mass index; CKD, chronic kidney disease; LAD, left atrial diameter; EF, ejection fraction; BNP, brain natriuretic peptide; CRP, C-reactive protein; eGFR, evaluated glomerular filtration rate; PGI2, prostacyclin*.

**Table 2:** Clinical characters of patients used for validation in this study.

	**SR (*n* = 18)**	**AF (*n* = 18)**	***P*-value**
Age (y)	55 ± 8	53 ± 8	0.565
Gender (Male/Female)	14/4	6/12	0.018
BMI (kg/m^2^)	23.7 ± 3.3	23.7 ± 4.1	0.999
**Cardiovascular risk factors**			
Hypertension (*n*)	10	4	0.086
Diabetes mellitus (*n*)	1	0	1.000
CKD (*n*)	1	0	1.000
Smoke abuse (*n*)	6	0	0.019
Alcohol abuse (*n*)	3	1	0.603
Coronary artery disease (*n*)	7	4	0.471
Stroke (*n*)	1	1	1.000
**Transthoracic echocardiography**			
LAD (cm)	4.7 ± 0.8	6.3 ± 1.6	<0.001
EF (%)	53.6 ± 10.2	50.6 ± 6.2	0.680
**Mitral valve Carpentier's phenotype (*****n*****)**			
II	11	2	
IIIa	1	11	
IIIb	5	14	
Mitral regurgitation with stenosis (*n*)	1	9	
**NYHA class (*****n*****)**			
II	8	0	
III	8	16	
IV	2	2	
BNP (pg/mL)	260.2 ± 413.3	330.7 ± 249.4	0.031
CRP (mg/L)	12.5 ± 30.0	11.2 ± 18.2	0.333
eGFR (%)	99.6 ± 13.9	96.7 ± 11.6	0.507
**Drug therapy (*****n*****)**			
Beta blockers	7	8	1.000
Anticoagulants	4	6	0.711
Digoxin	1	4	0.338
PGI2	4	5	1.000

*BMI, body mass index; CKD, chronic kidney disease; LAD, left atrial diameter; EF, ejection fraction; BNP, brain natriuretic peptide; CRP, C-reactive protein; eGFR, evaluated glomerular filtration rate; PGI2, prostacyclin*.

### RNA Preparation for RNA-seq

Total RNA was extracted from each sample using TRIzol Reagent (Life technologies, USA) according to the protocol from manufacturer. The concentration of each sample was measured by NanoDrop One (Thermo Scientific, USA). The quality was assessed by the Bioanalyzer 2200 (Agilent, USA). The RNA integrity was assessed by electrophoresis with denaturing agarose gel.

### mRNA, miRNA, and circRNA Library Preparation and Sequencing

The cDNA libraries of mRNA and circRNA were constructed for each RNA sample using the VAHTS^TM^ Total RNA-seq (H/M/R) according to the manufacturer's instructions. In summary, depletion of rRNA and fragmented into 150–200 bp using divalent cations at 94°C for 8 min. The cleaved RNA fragments were reverse-transcribed into first-strand cDNA, second-strand cDNA synthesis, fragments were end repaired, A-tailed and ligated with indexed adapters. Target bands were harvested through VAHTS^TM^ DNA Clean Beads. The products were purified and enriched by PCR to create the final cDNA libraries and quantified by Agilent 2200. The tagged cDNA libraries were pooled in equal ratio and used for 150 bp paired-end sequencing in a single lane of the Illumina HiSeq^TM^ 2500 with 51 plus seven cycles by NovelBio Corp. Laboratory, Shanghai.

The cDNA libraries of miRNA for single-end sequencing were prepared using Ion Total RNA-Seq Kit v2.0 (Life Technologies) according to the manufacturer's instructions. The cDNA library had been size selected by PAGE Gel electrophoresis for miRNA sequencing. The cDNA libraries were then processed for the Proton Sequencing process according to the commercially available protocols.

### RNA-seq Data Analysis

The EBSeq algorithm was used to filter differentially expressed circRNAs, miRNAs and mRNAs. After the significance analysis and FDR (false discovery rate) analysis, we selected the differentially expressed circRNA, miRNA and mRNA according to the threshold set at *P* < 0.05, FDR < 0.05, and fold change>1.5 or < -1.5. circRNAs were further filtered according to the following procedure: circRNAs which detected more than six samples in each group were retained, then DESeq2 algorithm (16) was used to perform the differential analysis. Finally, 177 circRNAs were remain for bioinformatics analysis. Volcano plot and gene heatmap were used to show the different circRNA expression pattern of PeAF and SR patients by R packages ggplot2 and pheatmap with R programming language (version 3.5.2).

### Construction of circRNA-miRNA-mRNA Network

To make a prediction downstream regulation of the circRNAs, we used the database of circAtlas 2.0 (http://circatlas.biols.ac.cn) to get a series of miRNAs. The database of PicTar (https://pictar.mdc-berlin.de), RNAhybrid (https://bibiserv.cebitec.uni-bielefeld.de/rnahybrid) and miRanda (http://www.microrna.org/microrna/home.do) were used to evaluate the possible binding sites between circRNAs and miRNAs. MiRNAs with more than two databases prompted and more than two binding sites existed would be compared with the results of the RNA-seq. Database of ENCORI (http://starbase.sysu.edu.cn) and TargetScan (http://www.targetscan.org/) were used to predict the downstream mRNAs. For each pair of RNAs, if the mRNA context score was < -0.95 and it appeared differentially expressed between SR and PeAF group (*P* < 0.05, FDR < 0.05 and fold change>1.5 or < -1.5), the paired miRNA and mRNA could be considered relevant. The Gene Ontology (GO) and Kyoto Encyclopedia of Genes and Genomes (KEGG) pathway enrichment analysis was performed to analyze the host genes and downstream genes of circRNAs. The clusterProfiler package (17) was used according to the manual. The circRNA-miRNA-mRNA network was built up by using Cytoscape version 3.5.1 (http://www.cytoscape.org).

### Validation of circRNAs With qRT-PCR and Sanger Sequencing

qRT-PCR was used to validate the differential expression of circRNAs identified by RNA-seq analysis in another 18 paired VHD patients with or without PeAF (primers of each circRNA were listed in Table 3). Total RNA was reverse transcribed to synthesize cDNA using PrimeScript RT reagent Kit (Cat. No RR047Q, Takara Biomedical Technology) and then analyzed by qRT-PCR with BrightGreen qPCR MasterMix (Cat. No MasterMix-S, abm Inc) with LightCycler 480 Instrument II (Roche Molecular System, Inc). Genomic DNA was excluded by using DNase enzyme during the reverse transcription. GAPDH was selected as the internal reference, the relative expression level of each circRNA was calculated using the 2^−ΔΔ*CT*^ method.

**Table 3:** Primer sequences of circRNA and internal reference used for validation by qRT-PCR.

**Primer Name**	**Sequences 5^**′**^to 3^**′**^**
circ 255-ITGA7 divergent-F	CCTATAATTGGAAGGACCTGTGC
circ 255-ITGA7 divergent-R	GCACAAAGCAGCGACCAATC
circ 418-KCNN2 divergent-F	CCGAGCTTGTGAAAGTTGTTC
circ 418-KCNN2 divergent-R	GGGCCGTCCATGTGAATGTA
circ 13913-MIB1 divergent-F	GATCACTGCCCTGTGCTAGG
circ 13913-MIB1 divergent-R	TGCTTGATGCCTATTGCCAC
circ 44670-BARD1 divergent-F	GGTGAACACCACCGGGTATC
circ 44670-BARD1 divergent-R	TCCTCCTAAACACACAGGCTC
circ 44782-LAMA2 divergent-F	TCCAGTAACAAGCTGGGACC
circ 44782-LAMA2 divergent-R	AGTTGAAATAGCCGTGGGCA
circ 81906-RYR2 divergent-F	GGAAGCTTCTTCAGCACTTGTC
circ 81906-RYR2 divergent-R	CTTCCACTCCACGCAACTCT
circ 35880-ANO5 divergent-F	AGCCATCAGAACCTCCCAATC
circ 35880-ANO5 divergent-R	AATCGCTTTGCAGGCATTGTT
circ 22249-TNNI3K divergent-F	GCCTCACTGCCCTCCATATT
circ 22249-TNNI3K divergent-R	AGACAGCCCATTTTCAGTGC
circ 3136-TNNI3K divergent-F	TATTGCAGTAGATGTTGCCAAAGG
circ 3136-TNNI3K divergent-R	CCAAGGGTGATGGAACAGACA
circ 56186-TNNI3K divergent-F	GCAGTGGGTCTCTCTCACCTTC
circ 56186-TNNI3K divergent-R	ATGCCCATCCTCATAGAGAAGAAT
GAPDH-F	GCCACATCGCTCAGACACC
GAPDH-R	CCCAATACGACCAAATCCGT

The backspliced junction sequence of each circRNA was amplified using Q5 high-fidelity DNA polymerase (Cat. No M0494S, NEB), the PCR products were then cloned into *pEASY*-Blunt Zero vector (Cat. No CB501-01, TransGen Biotech) according to the manufacturer's instructions. The following sanger sequencing was used to validate the junction sequence of individual circRNAs.

### Statistical Analysis

Numerical variables were described as mean ± standard deviation (SD), and the categorical variables were described as numbers or percentages. Differences between groups were assessed using Student's *t*-test or Wilcoxon's rank-sum test, as appropriate. Adjusted two-sided *P*-values were calculated and *P* < 0.05 was considered statistically significant. All statistical analyses were performed using SPSS 23 package (IBM, Chicago, IL, USA).

## Results

### Expression Profiles of Differentially Expressed circRNAs in Valvular AF LAA Tissue

**Figure 1:**
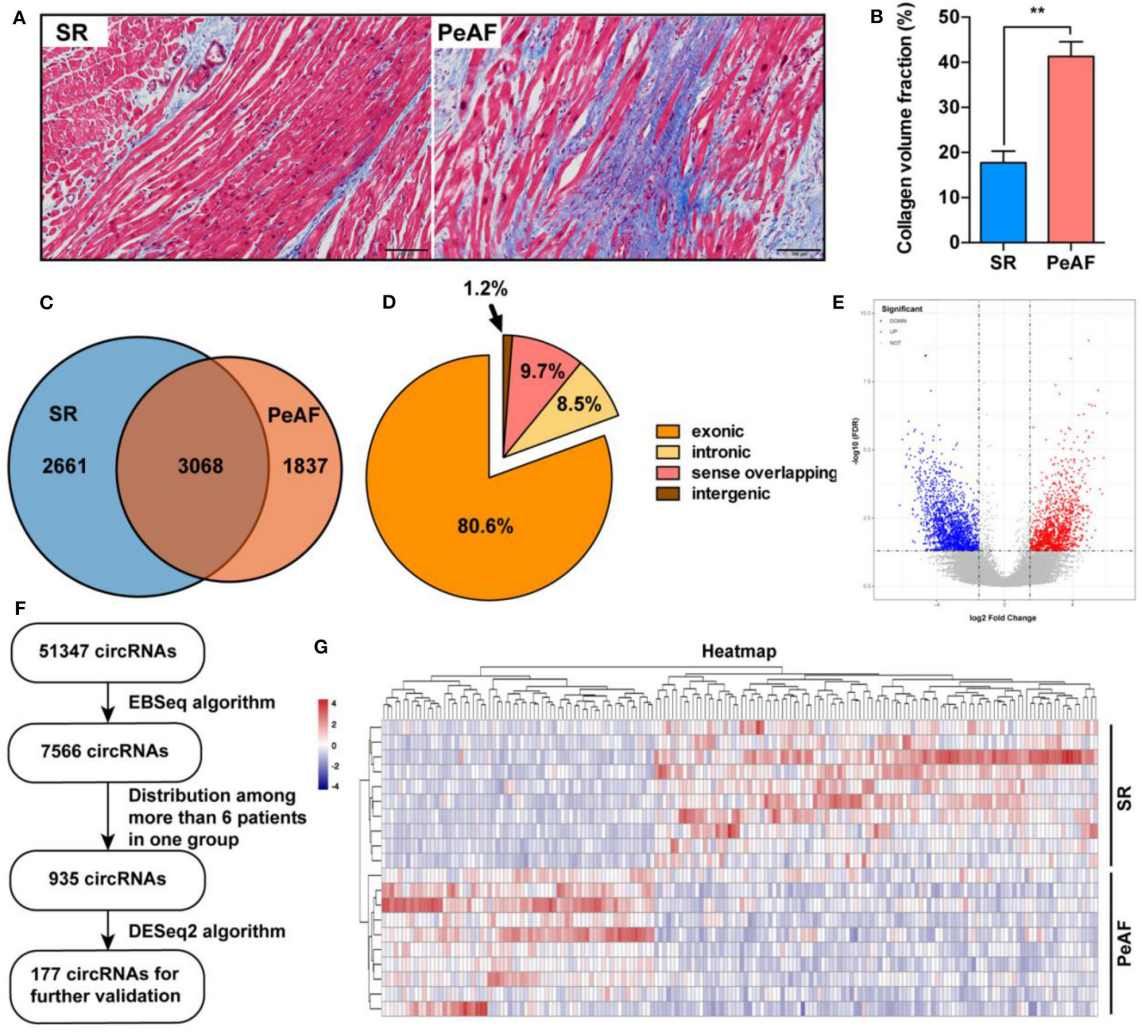
Expression profiles of circRNAs between SR and PeAF group. **(A)** Masson's staining of LAA in SR and PeAF patient. **(B)** Measurement of collagen volume fraction between SR and PeAF LAA tissues (17.7 vs. 41.3%, *P* < 0.01). **(C)** Venn diagram of differentially expressed circRNAs between SR and PeAF group. **(D)** Proportion of different types of circRNAs of RNA-Seq. **(E)** Volcano plot of differentially expressed circRNAs, red means upregulation, blue means downregulation. **(F)** Filter strategy diagram of differentially expressed circRNAs. **(G)** Heatmap of 177 differently expressed circRNAs between SR and PeAF group. ^*^p < 0.05; ^**^p < 0.01.

Ten paired LAA tissues of VHD patients with or without PeAF were used for RNA-seq. Before high throughput sequence, Masson staining was used to detect the ratio of fibrosis between two groups. Valvular PeAF patients showed more fibrotic area than SR group patients (17.7 vs. 41.3%, *P* < 0.01) (Figures 1A,B). In summary, A total of 51,347 circRNAs were detected, of which 7,566 circRNAs were significantly differentially expressed (*P* < 0.05, FDR < 0.05, fold change > 2) between two groups. 3,094 circRNAs were significantly up-regulated, and 4,472 circRNAs were significantly down-regulated in PeAF group (Figures 1C,E). Among all the differentially expressed circRNAs, 80.6% were exonic type, 8.5% were intronic type, 9.7% were sense overlapping type, the left 1.2% were intergenic type (Figure 1D). For further analysis of circRNAs in valvular PeAF, we made a filter of all differentially expressed circRNAs through a prearranged process and finally got 177 widely and differentitally expressed circRNAs (Figure 1F). A heatmap was conducted to visculize the circRNAs between SR and AF group (Figure 1G).

### Validation of Differentially Expressed circRNAs in LAA Tissues

**Figure 2:**
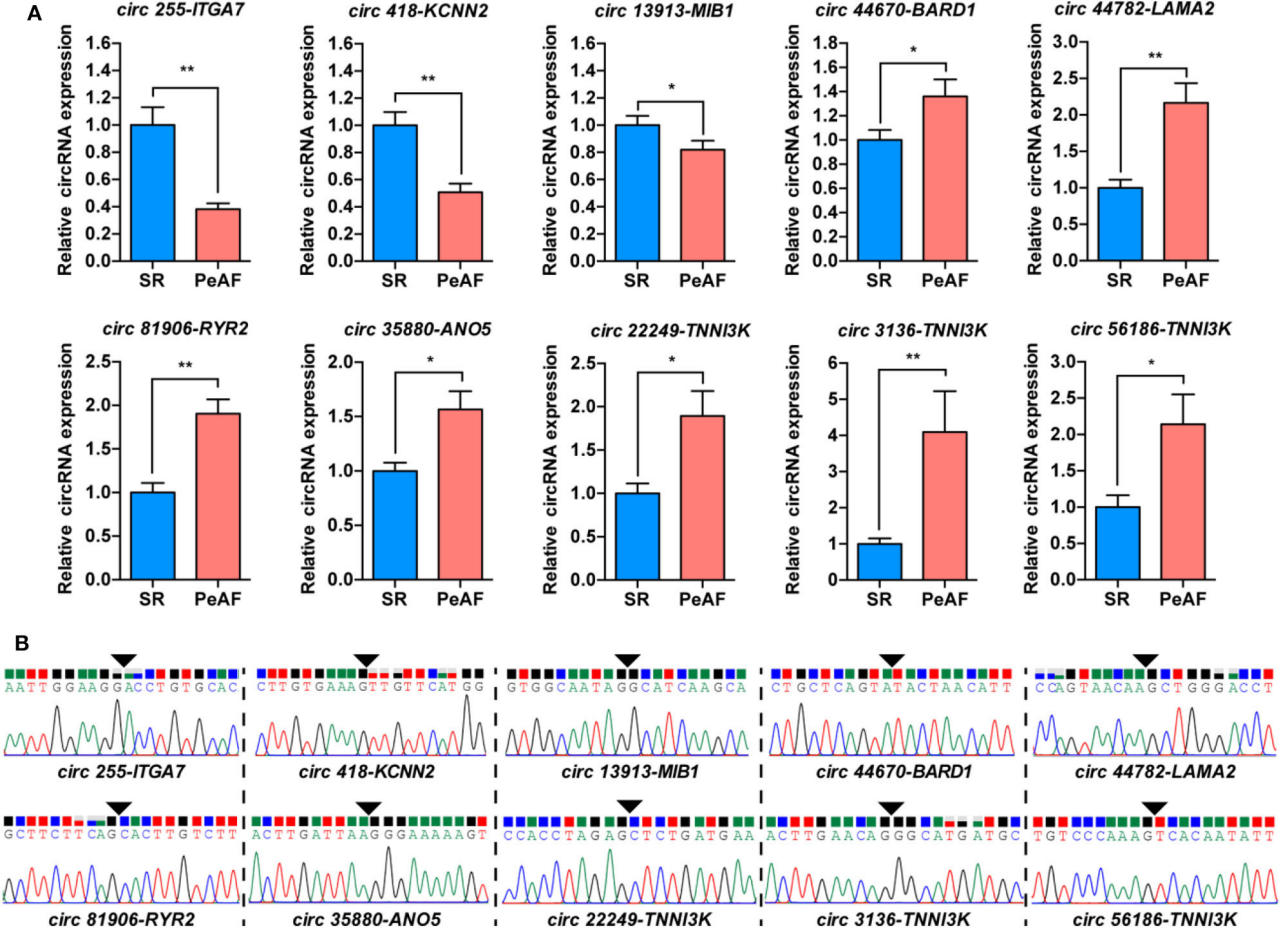
Validation of circRNAs between SR and PeAF group. **(A)** qRT-PCR results of selected circRNAs. circ 255-ITGA7, circ 418-KCNN2, and circ 13913-MIB1 were downregulated in PeAF group; circ 44670-BARD1, circ 44782-LAMA2, circ 81906-RYR2, circ 35880-ANO5, circ 22249-TNNI3K, circ 3136-TNNI3K, and circ 56186-TNNI3K were upregulated in PeAF group. **(B)** Sanger sequencing of backsplicing site of circRNAs selected for validation. Black triangle means backsplicing junction site. *p < 0.05; **p < 0.01.

The ten most differentially expressed circRNAs, which host genes were highly related with ion channel, myocardial sarcomere and focal adhesion were selected for subsequent verification by qRT-PCR. The expression of circ 255-ITGA7 (*P* < 0.0001), circ 418-KCNN2 (*P* = 0.0002) and circ 13913-MIB1 (*P* = 0.0444) were significantly downregulared in the valvular PeAF samples. The expression of circ 44670-BARD1 (*P* = 0.0394), circ 44782-LAMA2 (*P* < 0.001), circ 81906-RYR2 (*P* < 0.001), circ 35880-ANO5 (*P* = 0.0046), circ 22249-TNNI3K (*P* = 0.0487), circ 3136-TNNI3K (*P* = 0.0007) as well as circ 56186-TNNI3K (*P* = 0.0186) were upregulated obviously in valvular PeAF samples (Figure 2A). To further verify the reality of these circRNAs, the junction sequence of each circRNA was amplified with PCR, and the PCR products were then cloned into pEASY-Blunt Zero vector. Sanger sequencing was used to confirm the predicted head-to-tail back-splicing junction of these circRNAs (Figure 2B). These assays suggest that the overall false positive rate of the selected ten candidated circRNAs in our dataset is low.

### GO and KEGG Pathway Analysis of circRNAs Host Genes and Downstream Genes

**Figure 3:**
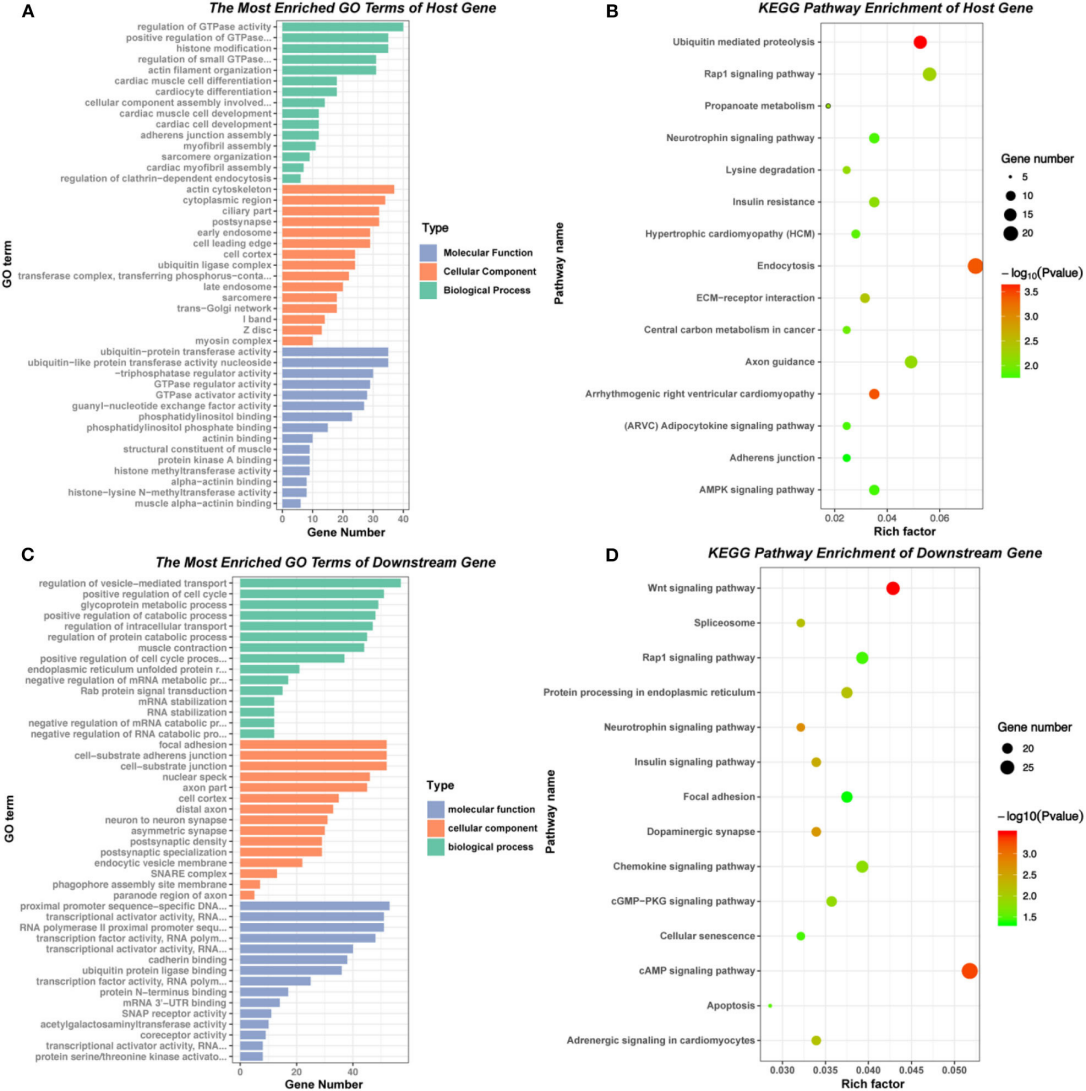
GO and KEGG pathway of host genes and downstream mRNAs of differentially expressed circRNAs. **(A)** and **(B)** GO and KEGG pathway analysis of the host genes of 177 differentially expressed circRNAs. **(C)** and **(D)** GO and KEGG pathway analysis of the downstream mRNAs in circRNA-miRNA-mRNA network.

The GO enrichment and KEGG pathway analysis of the host genes of differential expressed circRNAs were visualized in Figures 3A,B. These related host genes were mostly enriched in GTPase activity regulation, histone modification and actin filament organization in biological process (BP), actin cytoskeleton and cytoplasm in cellular component (CC), ubiquitin-protein transferase activity, GTPase regulator activity and guanyl-nucleotide exchange factor activity in molecular function (MF). In terms of pathway enrichment, host genes related signaling pathways mainly enriched in endocytosis, ubiquitin mediated proteolysis and Rap1 signaling pathway by KEGG analysis.

According to the basic function of circRNA, we further performed the GO and KEGG analysis of downstream genes of the six verified circRNAs (circ 255-ITGA7, circ 418-KCNN2, circ 13913-MIB1, circ 44782-LAMA2, circ 81906-RYR2, and circ 3136-TNNI3K). The results showed that most downstream genes enriched in regulation of vesicle-mediated transport, positive regulation of cell cycle and glycoprotein metabolic process in BP, focal adhesion, cell-substrate junction and nuclear speck in CC, transcriptional activator activity, cadherin binding and ubiquitin protein ligase binding in MF. KEGG pathway enrichment showed that the most related signaling pathways were cAMP signaling pathway and Wnt signaling pathway (Figures 3C,D, Supplementary Table 1).

### Prediction of circRNA-miRNA-mRNA Interaction Network

**Figure 4:**
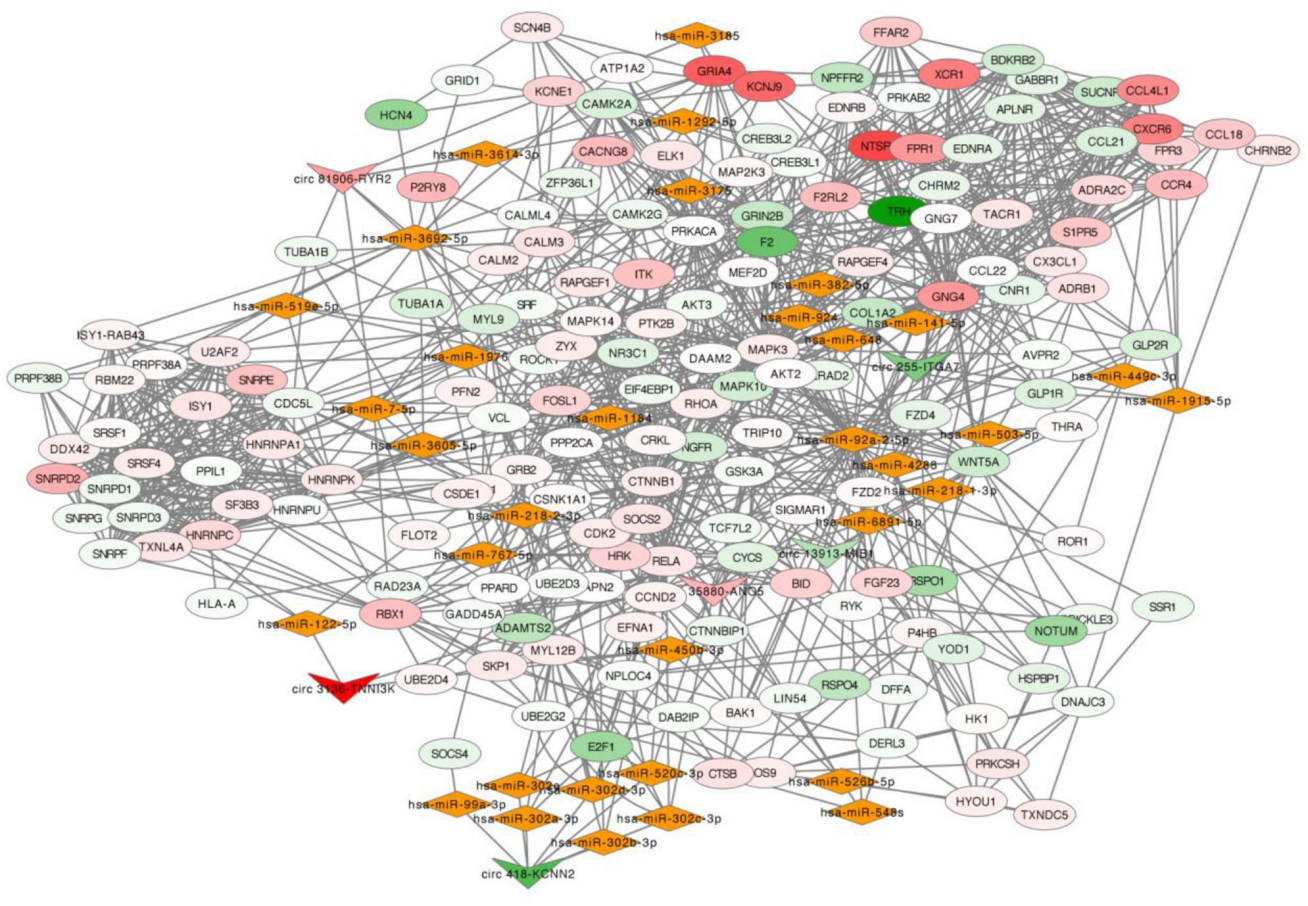
circRNA-miRNA-mRNA network. In this network, inverted triangle represents circRNA, rhombus means miRNA and ellipse means mRNA. The color intensity of circRNAs and mRNAs was related with the fold change of single gene in RNA-seq. Upregulation was visualized in red and downregulation in green.

The selected six dysregulated circRNAs with associated miRNAs and the downstream mRNAs were used to build up a circRNA-miRNA-mRNA interaction network (Figure 4). A single circRNA would affect several miRNAs and then induce multiple effects on the downstream mRNAs as an amplification of cascade reaction. For example, circ 418-KCNN2 would affect function of a miRNA cluster (hsa-miR-302a-3p, hsa-miR-302b-3p, hsa-miR-302c-3p, and hsa-miR-302d-3p) and then regulate the expression of RELA, also known as coding gene of transcription factor p65, by post-translational modification (Supplementary Figure 1). circ 81906-RYR2 would make an influence of the expression of intracellular calcium activity related genes CALM2 and CALM3 by regulating the function of hsa-miR7-5p (Supplementary Figure 1).

## Discussion

Valvular atrial fibrillation is a separate category of atrial fibrillation because of the progression of atrial lesion is different compared with the lone atrial fibrillation. Heart valve stenosis or regurgitation is the main cause of atrial structure remodeling due to atrial pressure or volume overload (18). Valve disease related chronic atrial stretch has been mentioned to be a high risk factor of the occurrence of AF. John et al. (19) have found that chronic atrial stretch would increase left atrial size, increase left atrial effective refractory period, increase low voltage area size and increase electrical silence area size in the meanwhile decrease left atrial conduction velocity in mitral stenosis induced AF patients. Chronic atrial stretch induced atrial dilation and atrial fibrosis will disrupt the electrical conduction into slow and discontinuous conduction which is thought to be a substrate for the development of AF (20). Although the electrical remodeling and structure remodeling will be found in both atrium, the influence is greater in the left atrium than the right atrium. So, in this study, we chose LAA to explore the differentially expressed circRNAs in VHD induced AF patients.

In the past two decades, different researches of AF animal models and patient samples have revealed different signaling pathway involved during the occurrence and the development of AF (21). Recent data also emphasized the importance of epigenetic mechanisms involved in AF, including DNA methylation (22, 23), histone modification and non-coding RNA regulation (24). CircRNA is a member of non-coding RNAs generated from back-splicing events between a downstream 3′ splicing site and an upstream 5′ splicing site of pre-messenger RNA. It has been considered to play important roles in the biological process of cancer (25), neurodegenerative diseases (26) and cardiovascular disease (27). We have found that there were some researches exploring the expression characterization of circRNA in atrial fibrillation. Zhang et al. (28) mentioned the expression profiles of circRNA in non-valvular persistent AF of four paired patient samples and found 296 differentially expressed circRNAs; Hu et al. (29) described the circRNAs in AF patients with rheumatic heart disease; Marina et al. (30) figured out a circRNA-miRNA cross-talk between paroxysmal and permanent AF. In this study, we revealed the differentially expressed circRNAs between VHD patients with or without AF in order to find the possible molecular targets during the progress of AF in the condition of atrial structure remodeling.

In our study, we found 7,566 differentially expressed circRNAs (*P* < 0.05, FDR < 0.05, fold change > 2) between the two groups. Three thousand and ninety four circRNAs were significantly up-regulated, and 4,472 circRNAs were significantly down-regulated in PeAF group. Only 177 most differentially expressed circRNAs were filtered for further validation through the gene filter process. Ten candidate circRNAs were found significantly differentially expressed in another 18 pairs of patients' samples between AF and SR group. Sanger sequencing was conducted to verify the back-splicing site of specific circRNAs. Furthermore, a circRNA-miRNA-mRNA network was built up to predict the circRNA-miRNA-mRNA axis involved in the occurrence of AF in VHD.

The most studied function of circRNA is acting as a strong miRNA sponge to regulate the biological function of downstream miRNAs and affect the miRNA-targeted mRNAs in an indirect post-transcriptional signaling pathway (31). We believe that it is useful to conduct a network of circRNA, miRNA and mRNA to predict the possible signaling pathway for further analysis of atrial fibrillation. The miRNA and mRNA sequencing data were originated from the same samples used in this study. Six verified circRNAs (circ 255-ITGA7, circ 418-KCNN2, circ 13913-MIB1, circ 44782-LAMA2, circ 81906-RYR2, and circ 3136-TNNI3K) were selected for further network construction. Database including circAtlas 2.0, ENCORI, TargetScan, miRanda, PicTar, and RNAhybrid combining the RNA-seq results of miRNA and mRNA were used to construct the gene regulatory network. circ 81906-RYR2 was found highly expressed in PeAF patients and would regulate the expression of CALM2 and CALM3 through regulating function of miR-7-5p, which were involved in cAMP signaling pathway, Rap1 signaling pathway, cGMP-PKG signaling pathway, adrenergic signaling. CALM2 and CALM3 are the mainly coding gene of calmodulin (CaM) and the expression ratio of CALM2 and CALM3 in cardiomyocyte is 2:5 (32). Previous researches have showed that mutation of CALM2 or CALM3 was the main reason for catecholamine-sensitive ventricular tachycardia and long QT syndrome (33–35). *In vitro* experiment also found the increasing expression of CaM in AF cell model (36). Therefore, circ 81906-RYR2/miR-7-5p/CALM2/3 axis needs further exploration for the pathological process of AF occurrence in VHD patients. We also found that a single circRNA could affect several miRNAs and then induce multiple effects on the downstream mRNAs as an amplification of cascade reaction. circ 418-KCNN2 could affect function of a miRNA cluster (hsa-miR-302a-3p, hsa-miR-302b-3p, hsa-miR-302c-3p and hsa-miR-302d-3p) and then regulate the same downstream gene RELA which is also known as the coding gene of p65. Recent animal study revealed that p65 NF-κB signaling may contribute to the inflammatory reaction in the pathogenesis of AF (37).

Several limitations of this study should be mentioned. First, in this study, we only verified the top 10 circRNAs of which the host genes were related with ion channel, myocardial sarcomere and focal adhesion, still a lot of differentially expressed circRNA needed to be verified. Second, in this study, we only verified the back-splicing site of specific circRNA by using divergent primers of cDNA sample. Genomic DNA and convergent primers are also needed for the complete verification. Third, although we use a set of filter method to get the more accurate results, the sample size of this study is still limited which will increase the false positive rate during analyzing data of RNA-Seq. Moreover, more further experiments are needed to explore the regulatory effects of circRNAs in the occurrence of AF in VHD patients.

## Conclusions

In summary, this study revealed the differentially expressed circRNAs between VHD patients with or without AF in order to find the possible molecular targets during the progression of AF in the condition of atrial structure remodeling. Thousands and hundreds circRNAs were found to take participate during the disease process. Among them, a set of ten differentially expressed circRNAs was verified, and six of them were used to construct a circRNA-miRNA-mRNA network. Multiple famous signaling pathways have been figured out for further study, such as cAMP signaling pathway and Wnt signaling pathway. This study provided a preliminary landscaping of circRNAs expression profiles which are involved in persistent AF due to VHD, and established the probability for future related researches in this field.

## Data Availability Statement

Raw data of this study is available in the database of SRA project No. PRJNA667522, and the expression matrix of circRNAs in each sample is available in Supplementary Material.

## Ethics Statement

The studies involving human participants were reviewed and approved by Medical Ethics Committee of Nanjing Drum Tower Hospital, the affiliated hospital of Nanjing university medical school (approval number: 2016-151-01). The patients/participants provided their written informed consent to participate in this study.

## Author Contributions

XZ analyzed data of the RNA-Seq and wrote the manuscript. XT and HCh helped make the vector construction. HCa, FF, and JP collected the tissue samples. DW designed the research. QZ made the revision of the manuscript and designed the research. All authors contributed to the article and approved the submitted version.

## Conflict of Interest

The authors declare that the research was conducted in the absence of any commercial or financial relationships that could be construed as a potential conflict of interest.
