# Supplementary material for: Expression Profiles of Circular RNA in Human Atrial Fibrillation With Valvular Heart Diseases
Source: Front Cardiovasc Med. 2020 Nov 20;7:597932. doi: 10.3389/fcvm.2020.597932 (PMC7714832; doi:10.3389/fcvm.2020.597932)
Supplement: Supplementary file 1 [file Table_1.DOCX]

Supplementary Material

# Supplementary figure


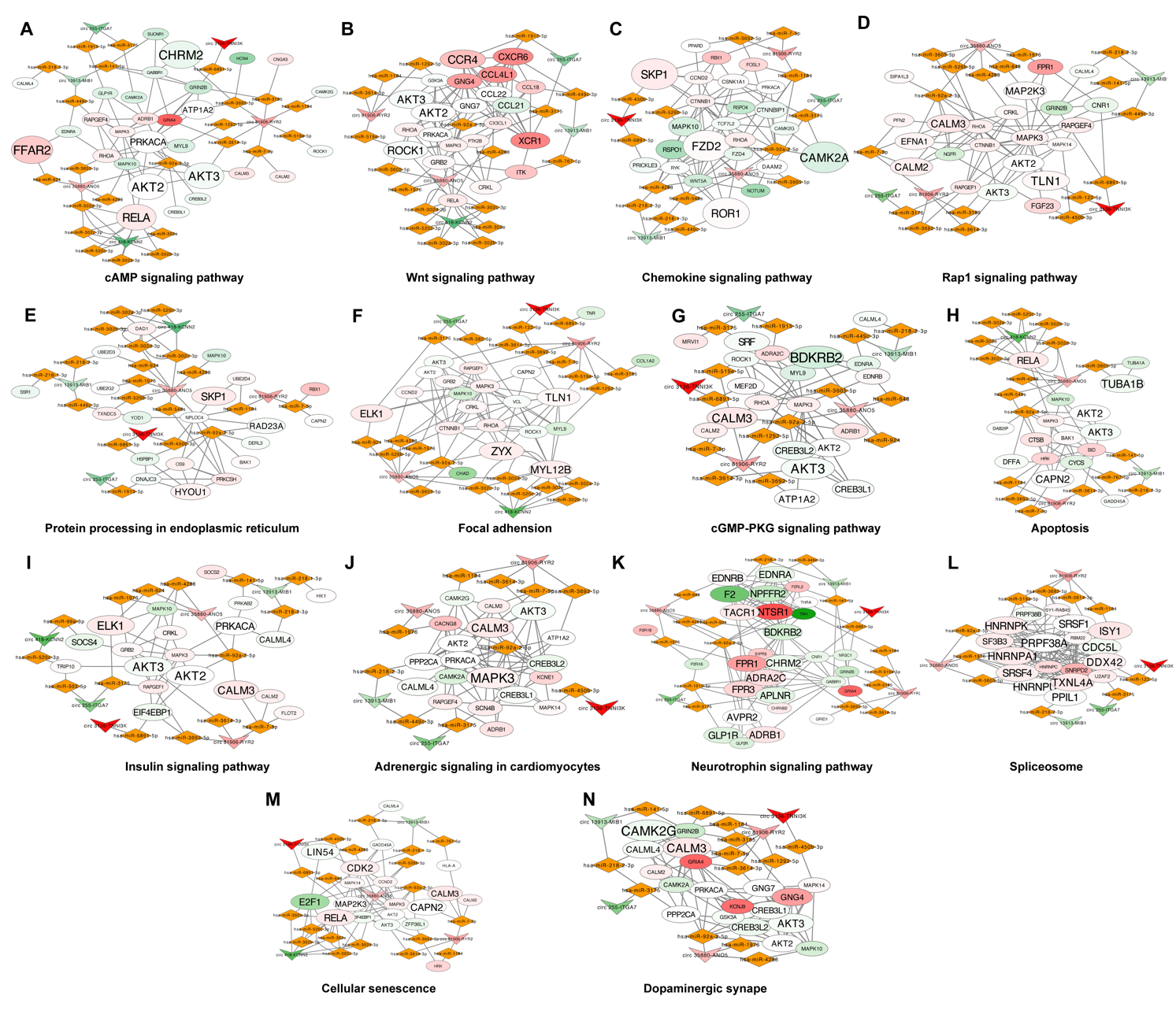


**Supplementary Figure 1. Top 14 signaling pathway networks consist of circRNAs, miRNAs and mRNAs.** **(A)** cAMP signaling pathway **(B)** Wnt signaling pathway **(C)** Chemokine signaling pathway **(D)** Rap1 signaling pathway **(E)** Protein processing in endoplasmic reticulum **(F)** Focal adhension **(G)** cGMP-PKG signaling pathway **(H)** Apoptosis **(I)** Insulin signaling pathway **(J)** Adrenergic signaling in cardiomyocytes **(K)** Neurotrophin signaling pathway **(L)** Spliceosome **(M)** Cellular senescence **(N)** Dopaminergic synape.


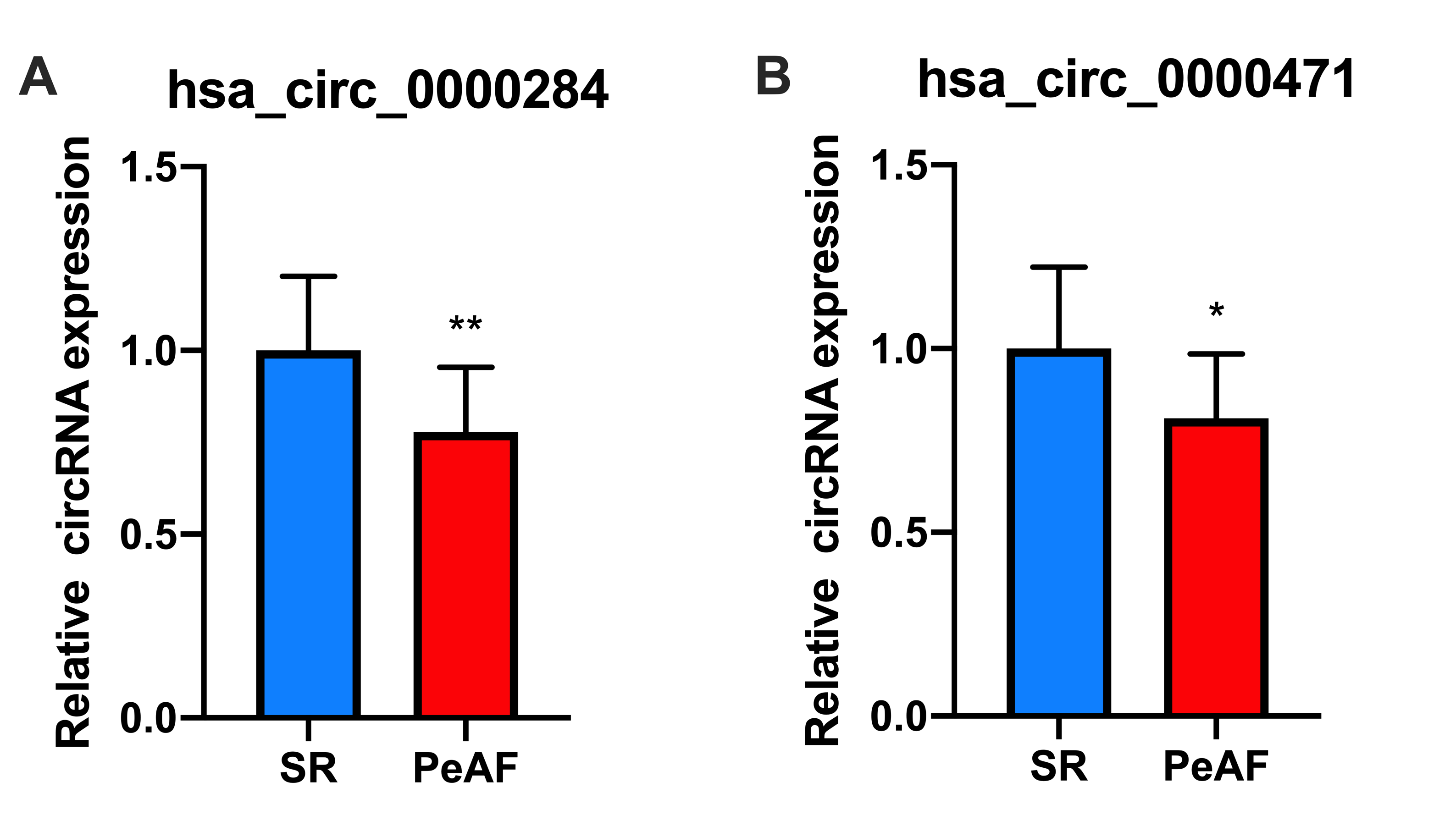


**Supplementary Figure 2. Expression of hsa_circ_0000284 and hsa_circ_0000471 between SR and AF group. (A) The expression of hsa_circ_0000284 was slightly decreased in AF group, P=0.0035. (B) The expression of hsa_circ_0000471 was slightly decreased in AF group, P=0.022.**

# Supplementary Tables

**Supplementary Table 1.** KEGG pathway enrichment of downstream mRNAs.

|  | ID | Description | GeneRatio | BgRatio | pvalue | p.adjust | qvalue | Count | Rich factor |
| --- | --- | --- | --- | --- | --- | --- | --- | --- | --- |
| hsa04024 | hsa04024 | cAMP signaling pathway | 29/560 | 212/7844 | 0.00049746 | 0.0495805 | 0.04398622 | 29 | 0.05178571 |
| hsa04310 | hsa04310 | Wnt signaling pathway | 24/560 | 158/7844 | 0.00032179 | 0.04810759 | 0.0426795 | 24 | 0.04285714 |
| hsa04062 | hsa04062 | Chemokine signaling pathway | 22/560 | 190/7844 | 0.0162583 | 0.22096505 | 0.19603307 | 22 | 0.03928571 |
| hsa04015 | hsa04015 | Rap1 signaling pathway | 22/560 | 206/7844 | 0.03682075 | 0.27523507 | 0.24417968 | 22 | 0.03928571 |
| hsa04141 | hsa04141 | Protein processing in endoplasmic reticulum | 21/560 | 165/7844 | 0.00670644 | 0.13271451 | 0.11774003 | 21 | 0.0375 |
| hsa04510 | hsa04510 | Focal adhesion | 21/560 | 199/7844 | 0.04537439 | 0.29493352 | 0.26165551 | 21 | 0.0375 |
| hsa04022 | hsa04022 | cGMP-PKG signaling pathway | 20/560 | 166/7844 | 0.01432637 | 0.21464644 | 0.1904274 | 20 | 0.03571429 |
| hsa04728 | hsa04728 | Dopaminergic synapse | 19/560 | 131/7844 | 0.00228324 | 0.08533605 | 0.07570739 | 19 | 0.03392857 |
| hsa04910 | hsa04910 | Insulin signaling pathway | 19/560 | 137/7844 | 0.0038158 | 0.1059371 | 0.09398398 | 19 | 0.03392857 |
| hsa04261 | hsa04261 | Adrenergic signaling in cardiomyocytes | 19/560 | 145/7844 | 0.00710178 | 0.13271451 | 0.11774003 | 19 | 0.03392857 |
| hsa04722 | hsa04722 | Neurotrophin signaling pathway | 18/560 | 119/7844 | 0.00183305 | 0.08533605 | 0.07570739 | 18 | 0.03214286 |
| hsa03040 | hsa03040 | Spliceosome | 18/560 | 134/7844 | 0.00671135 | 0.13271451 | 0.11774003 | 18 | 0.03214286 |
| hsa04218 | hsa04218 | Cellular senescence | 18/560 | 160/7844 | 0.03593134 | 0.27523507 | 0.24417968 | 18 | 0.03214286 |
| hsa04210 | hsa04210 | Apoptosis | 16/560 | 136/7844 | 0.03243381 | 0.26859863 | 0.23829204 | 16 | 0.02857143 |
| hsa04916 | hsa04916 | Melanogenesis | 15/560 | 101/7844 | 0.00508894 | 0.11704551 | 0.103839 | 15 | 0.02678571 |
| hsa04114 | hsa04114 | Oocyte meiosis | 15/560 | 125/7844 | 0.03223802 | 0.26859863 | 0.23829204 | 15 | 0.02678571 |
| hsa04140 | hsa04140 | Autophagy - animal | 15/560 | 128/7844 | 0.03868329 | 0.28210494 | 0.2502744 | 15 | 0.02678571 |
| hsa04670 | hsa04670 | Leukocyte transendothelial migration | 14/560 | 112/7844 | 0.02780692 | 0.26625746 | 0.23621503 | 14 | 0.025 |
| hsa04919 | hsa04919 | Thyroid hormone signaling pathway | 14/560 | 116/7844 | 0.03616539 | 0.27523507 | 0.24417968 | 14 | 0.025 |
| hsa04912 | hsa04912 | GnRH signaling pathway | 13/560 | 93/7844 | 0.01435762 | 0.21464644 | 0.1904274 | 13 | 0.02321429 |
| hsa04713 | hsa04713 | Circadian entrainment | 13/560 | 97/7844 | 0.01989654 | 0.24787767 | 0.21990908 | 13 | 0.02321429 |
| hsa04066 | hsa04066 | HIF-1 signaling pathway | 13/560 | 100/7844 | 0.02500319 | 0.26625746 | 0.23621503 | 13 | 0.02321429 |
| hsa04625 | hsa04625 | C-type lectin receptor signaling pathway | 13/560 | 104/7844 | 0.03323796 | 0.26859863 | 0.23829204 | 13 | 0.02321429 |
| hsa04668 | hsa04668 | TNF signaling pathway | 13/560 | 110/7844 | 0.04900302 | 0.31174261 | 0.27656799 | 13 | 0.02321429 |
| hsa04350 | hsa04350 | TGF-beta signaling pathway | 12/560 | 92/7844 | 0.02985604 | 0.26625746 | 0.23621503 | 12 | 0.02142857 |
| hsa05031 | hsa05031 | Amphetamine addiction | 11/560 | 68/7844 | 0.00820217 | 0.1442617 | 0.12798433 | 11 | 0.01964286 |
| hsa04917 | hsa04917 | Prolactin signaling pathway | 10/560 | 70/7844 | 0.02595884 | 0.26625746 | 0.23621503 | 10 | 0.01785714 |
